# Long-Term Functional Results of Intra- and Extra-Articular Hand Fractures Treatment With Titanium Miniature Plates and Screws With Minimum Follow-Up of 24 Months

**DOI:** 10.7759/cureus.15438

**Published:** 2021-06-04

**Authors:** Sokratis Varitimidis, Zoe Dailiana, Dimitrios Agorastakis, Apostolos Fyllos, Aristeidis Zibis, Michael Hantes, Konstantinos Malizos

**Affiliations:** 1 Orthopaedics and Musculoskeletal Trauma, Faculty of Medicine, University Of Thessaly, Larissa, GRC; 2 Orthopaedics and Musculoskeletal Trauma, Faculty of Medicine, University of Thessaly, Larissa, GRC; 3 Anatomy, School of Health Sciences, University of Thessaly, Larissa, GRC; 4 Orthopedics and Musculoskeletal Trauma, Faculty of Medicine, University of Thessaly, Larissa, GRC

**Keywords:** metacarpal fractures, phalangeal fractures, plates, screws, hand fractures, trauma, internal fixation

## Abstract

Introduction

The purpose of this study was to evaluate the long-term outcome of an extended range of hand fractures treated with titanium, low profile plates, and screws.

Materials and methods

This retrospective study included adult patients with at least one phalangeal and/or metacarpal fracture, treated with mini titanium plates and screws, between 2004-2016, in a single trauma center, that were followed-up for at least 24 months and with complete, intact medical records.

Results

90 patients (79 men and 11 women) with 114 hand (46 phalangeal, 68 metacarpal) fractures fulfilled the inclusion criteria. Thirty-two fractures were open (28.07%), 27 were intra-articular (23.68%), and 12 were both open and intra-articular fractures. The mean age of the patients was 36.02 years (range 17-75). Mean follow-up was 95.3 months (range 24 to 138). Open fractures had a reduced mean grip strength and total active motion. No difference was observed between intra-articular and extra-articular fractures (for grip strength and total active motion). Predictors of the final outcome included the severity of the initial injury (open vs closed) and not the anatomic location (intra- or extra-articular, metacarpal, or phalangeal) of the fracture.

Conclusions

Low-profile plates and screws can successfully be used to establish union and restore the alignment of the fractured bone while achieving a satisfactory clinical outcome, even in cases of open or intra-articular fractures.

## Introduction

About 20% of all fractures in the human skeleton involve the hand, making them less common only to forearm fractures, and affecting primarily the young, active population [1]. The decision about the method of treatment should take into consideration the specific anatomy of each bone and the importance of restoration of hand function, individualized according to patient needs and wishes. Surgical treatment is reserved for unstable, irreducible, comminuted, intra-articular, and open fractures [2].

Popular surgical techniques include fixation with Kirschner wires (K/Ws) [3-6] and mini or dynamic external fixators [7-9]. Mini plates and screws [4,10-21] offer, however, a more rigid fixation without compromising functional outcome [5,22]. Early designs of plates and screws for the treatment of unstable fractures were large and cumbersome compared to the fine anatomy of the hand [12]. Newer low-profile implants were developed, which offer periosteal closure in order to overcome the problem of adhesions and offer better functional results [23,24]. Furthermore, bio-absorbable implants were designed and used for the treatment of these fractures as well [11,14,19,20]. The choice is a matter of surgeon familiarity, the personality of the fracture, and the local tissue environment.

The purpose of this retrospective case series was to evaluate the long-term clinical and functional outcomes (including surgical technique and complications) of a wide range of hand fractures (closed or open, intra- or extra-articular) surgically treated with low profile mini plates and screws or screws alone, in a single hand-trauma center.

This paper was previously presented as an abstract titled "A-0765 The use of titanium miniature plates and screws for the treatment of intra and extra-articular fractures of the hand" at the XXIII Federation of European Societies for the Surgery of the Hand (FESSH) Congress held in Copenhagen, Denmark from 13-16 June 2018.

## Materials and methods

We retrospectively reviewed case files of patients that suffered a hand fracture and were treated with mini titanium plates and screws in a single hand-trauma center. Ethical approval for this study was obtained from the Institutional Ethics and Scientific committee (08/01/2020, ID:814). Inclusion criteria: Adult patients with at least one phalangeal and/or metacarpal fracture, treated with mini titanium plates and screws, between 2004-2016, that were followed-up for at least 24 months and with complete medical records. Demographics, type of labour (manual or desk job), hand dominance, fracture type, mean time from injury to surgery, type of anesthesia, and postoperative clinical scores were analysed. The patients were reviewed in the outpatient clinic every three weeks during the first three months and then at six, 12, 18, and 24 months, and annually after the last follow-up. Pain was evaluated with the Visual Analog Scale and function was measured with the Disability of the Arm Shoulder and Hand (DASH) Greek questionnaire [25]. Patient satisfaction was assessed on a scale of 0 to 10 (0 not satisfied at all to 10 maximum satisfaction). During clinical examination, total active range of motion (TAM) and tip to palm distance in the injured ray was measured in each patient, while tip pinch and grip strength was compared to the contralateral hand. Patients that suffered from amputation were excluded from these measurements. Results from the last recorded follow-up are presented.

Statistical analysis was performed with SPSS version 13.0 software (SPSS Inc., Chicago, USA). Grip strength and TAM was compared between open and closed fractures, intra- and extra-articular, metacarpal, and phalangeal fractures with the independent samples Student’s t-test.

## Results

Out of 121 patients treated for this condition, 90 patients (79 men and 11 women) with 114 hand fractures fulfilled all the inclusion criteria. The mean age of the patients was 36.02 years (range 17-75, SD 7.9). The majority of patients (63%) consisted of manual workers. The dominant hand was affected in 55% of the patients. The mean follow-up was 95.3 months (range 24 to 138, SD 10.1). 

There were 46 phalangeal and 68 metacarpal fractures in the cohort and involved 113 rays. The distribution of fractures by location and type is depicted in Tables 1, 2. Thirty-two fractures were open (28.07%), 27 were intra-articular (23.68%), and 12 were both open and intra-articular fractures. Twenty-three of the 32 open fractures involved combined hand injuries, and in 11 cases, open fractures were associated with incomplete (six) or complete (five) amputations (severe damage to one or both neurovascular bundles, respectively). In one patient, there was a pathological fracture due to a metacarpal enchondroma. Intra-articular fractures involved one side of the joint in 25 patients, while two intra-articular fractures were on opposite sides of the same joint. These were measured as two different fractures rather than one. All fractures were operated with the Leibinger low-profile titanium plates and screws system (Stryker Leinbinger Inc., Kalamazoo, MI, USA).

**Table 1:** Distribution of surgically treated hand fractures (total of 114 fractures).

	Thumb	Index	Middle	Ring	Little
Metacarpal	5	14	14	13	22
P1	5	3	7	6	12
P2	1	4	4	3	1
P3		0	0	0	0

**Table 2:** Distribution of open/closed and intra-/extra-articular fractures in the metacarpal and phalangeal bones (total of 114 fractures).

	Metacarpal fractures		Phalangeal fractures		
	Intra-articular	Extra-articular	Intra-articular	Extra-articular	Total
Open	4	13	8	7	32
Closed	7	44	8	23	82
Total	11	57	16	30	114

The fractures were fixed after a mean of 2.9 days (range 0-28) from injury. Regional anaesthesia was utilized in 85 patients and only five patients received general anaesthesia. The reason for using general anaesthesia in these patients was the need to fix other fractures (in the lower extremity) in the same anaesthetic session (four cases), whereas in one case, complete amputation of five digits of the same hand was needed. Several different types of low-profile plates (straight, L shaped, and H shaped) were used for 85 fractures according to the location and the specific morphology of the fracture. In the remaining 29 fractures, internal fixation was performed only with screws. Fixation with screws was selected for condylar or intra-articular fractures of the base of metacarpals and phalanges, and for long oblique or spiral diaphyseal fractures. For these diaphyseal fractures, the length of the fracture line had to be at least 2.5 times the diameter of the diaphysis and was fixed with at least two lag screws.

Anatomic reduction of the fractures was achieved in all cases except one, in which a primary arthrodesis of the proximal interphalangeal (PIP) joint had to be performed because of severe comminution on both sides of the PIP joint. Fluoroscopy was utilized in all cases to anatomically reduce the fractures.

Mean operative time for simple fractures treated with open reduction and internal fixation (ORIF) with screws was 43.53 minutes (range 34-65, SD 6.9) and was significantly shorter (p=0.009) than for those treated with ORIF with both plates and screws for which the mean time was 46.9 minutes (range 38-70, SD 9.2). Data on the operative time from patients with complex injuries that required extensive soft tissue reconstruction or with concomitant injuries were not included. Bone graft was used in one case. This patient had an open intra-articular metacarpal fracture with comminution and an effort was made to restore the joint with the use of a low-profile plate, a K/W, and bone graft. In three other fractures, K/Ws were also employed together with plates or screws.

Postoperatively, antibiotic prophylaxis was administered according to institutional protocol for 24 hours in case of closed fractures. In open fractures, intravenous antibiotics with tetanus prophylaxis were used as soon as possible after patient arrival and antibiotics were continued for three to five days depending on the estimated bacteriological burden of the wound. After tendon repair, a splint was applied. Hand elevation for 24 to 48 hours and finger motion was encouraged for edema control. Patients with closed fractures were discharged the next day after dressing change. Hospital stay was extended appropriately for patients with open fractures, crush injuries, and amputations. Physical therapy in the outpatient clinic was individualized.

**Figure 1:**
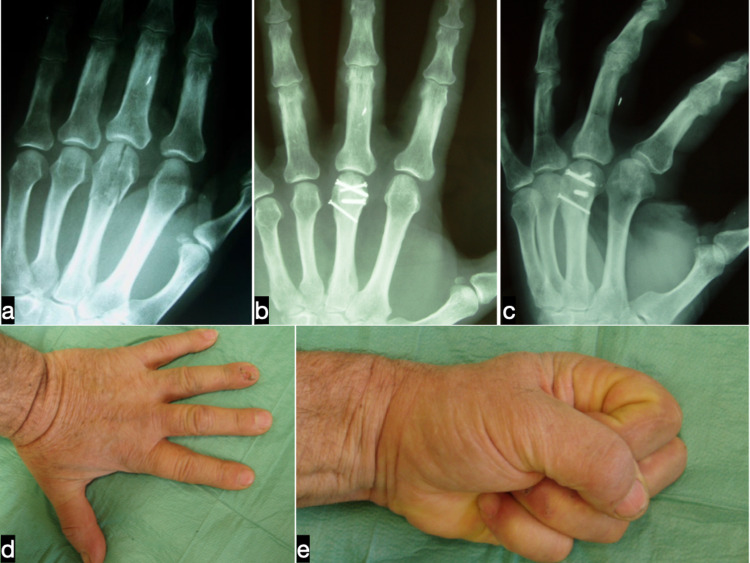
Preoperative (a) anteroposterior radiograph of a 64-year old male carpenter who sustained an open intra-articular fracture of the third metacarpal head of his non-dominant hand. The fracture was treated with four titanium mini screws in an effort to restore the joint line. Radiographic (b,c) and clinical (d,e) outcomes seven years postoperatively.

**Figure 2:**
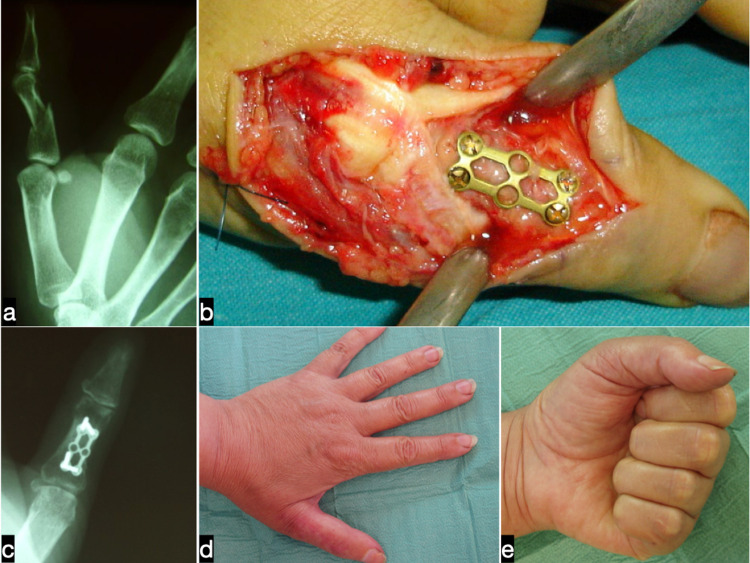
Preoperative (a) anteroposterior radiograph of a closed extraarticular fracture of the proximal phalanx of the dominant thumb in a 49-year old female patient. Intraoperative view of the fracture fixation with the use of a titanium H-plate (b). Radiographic (c) and clinical (d,e) outcome, six years postoperatively

**Figure 3:**
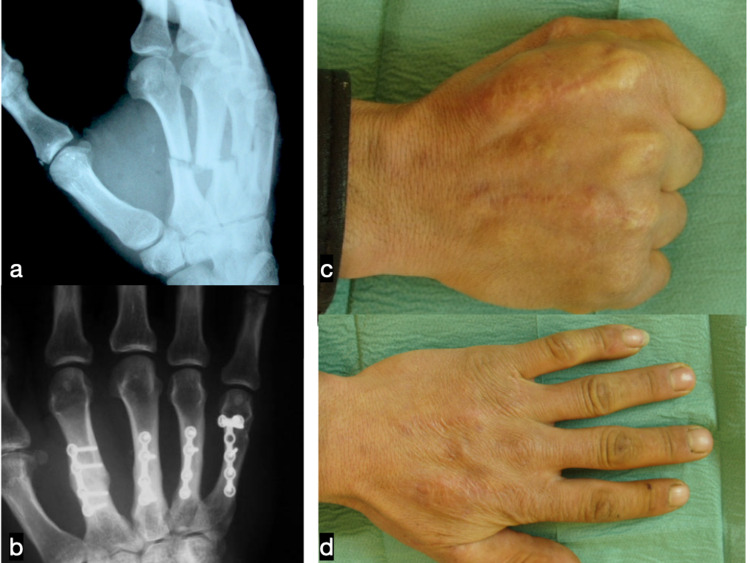
Preoperative radiograph of a patient who sustained closed fractures of the second through the fifth metacarpal (a). He underwent plate fixation of all fractures (b). Range of motion is presented six years after treatment (c,d)

The mean time to fracture healing was 5.3 weeks (range 4-9 weeks). Clinical results are summarized in Table 3 and three clinical cases are depicted in Figures 1-3. At the final follow up, the mean grip strength was measured as high as 89% compared to the uninjured hand even in cases of intra-articular fractures (Table 3). Open intra-articular fractures had the worst outcome in grip strength (67% to the contralateral hand). With regards to pinch strength, open intra-articular fractures once again were the fractures group with the least favorable outcome (68% of the contralateral pinch strength). The DASH score was 8.7 (SD 4.5) in the intra-articular fractures group and reached 15.6 (SD 9.1) in the open intra-articular fractures group. The mean patient satisfaction ranged from 7 to 10 across all fracture groups. Finally, everyday pain in the last month as measured with the Visual Analog Scale reached a maximum of 2.3 in all groups, except for open intra-articular fractures, which demonstrated a mean of 3.2.

**Table 3:** Results according to the type of the original injury (total of 114 fractures). Grip and tip pinch strengths are presented as percentages (%) of the strengths of the uninjured side. Two patients suffered from both closed intra- and extra-articular fractures of different hand rays. * TAM for the thumb fractures is given in parentheses. TAM: total active range of motion; VAS: Visual Analog Scale; DASH: Disability of the Arm Shoulder and Hand.

	Intra-articular			Extra-articular		
	total (27 in 25 patients)	open (12 in 11 patients)	closed (15 in 14 patients)	total (87 in 67 patients)	open (20 in 12 patients)	closed (67 in 55 patients)
Grip strength	89%	67%	105.2%	93.6%	98.3%	92.6%
Pinch strength	84%	68.2%	95.8%	93.2%	93.3%	93.2%
Tip to palm distance (mm)	5.4	11.6	0.83	0.59	0.25	0.7
TAM* (^o^)	216 (135)	203	228 (135)	215 (94)	198 (75)	219 (105)
DASH	8.7	15.6	3.7	3.8	7	2.8
Satisfaction	7.7	7	8.85	9.3	10	9
VAS	1.5	3.2	0.3	0.46	2.3	1.5

Grip strength and TAM were compared in open and closed fractures, in intra- and extra-articular fractures, and in metacarpal and phalangeal fractures. There was no statistically significant difference in grip strength (p=0.25) or TAM (p=0.849) between extra and intra-articular fractures (Table 3). TAM and grip strength were statistically different between open and closed fractures (p=0.001 for TAM and p=0.013 for grip strength respectively). Treatment of metacarpal and phalangeal fractures resulted in similar outcomes as far as TAM and grip strength is concerned. Implant removal due to patient wishes or hardware irritation was not deemed necessary in any patient, except for a malunion case described below.

Twelve complications were encountered in this study. These included superficial skin necrosis in three cases with an open extra-articular fracture and in one patient with an open intra-articular fracture after severe crush injuries of the fractured digits. These patients had one or both their digital arteries reconstructed during the fracture fixation. Superficial skin necrosis was managed with pedicled flaps in three patients, while one case ended up in partial amputation after two failed attempts trying to salvage the digit. Rotational malunion was observed in one case of a closed oblique diaphyseal fracture of the second metacarpal that was treated with a low-profile plate. In this patient, the plate had to be removed and a new plate was placed after rotational osteotomy of the metacarpal. Another five patients had complete or partial loss of tactile sensation on either side of the injured digit. One or both digital nerves were damaged in the initial injury in all five cases but an attempt for reconstruction was made in only three cases. Final outcome was considered satisfactory in all patients and no other reconstructive procedures were performed. Finally, two patients developed complex regional pain syndrome and were treated with intravenous regional anesthesia (Bier's block) using xylocaine and methylprednisolone with the diminishing of the symptoms after one month in both patients.

## Discussion

In this study, we evaluated and presented the outcome of low-profile titanium implants in a wide range of metacarpal and phalangeal fractures. A broad variety of fractures were managed with the low-profile system, ranging from simple, closed, uncomplicated fractures to complex, open, intra-articular fractures. Given that there is a large difference in the fracture patterns and the underlying mechanisms, it is difficult to perform focused and controlled research. Rather than focusing on a specific fracture type or patient population, with the long period of follow-up and many clinical and functional parameters evaluated, this study gives a comprehensive insight into the application of these implants on a wide range of hand fractures. Patient-reported outcomes and pain measurement demonstrated that those implants are appropriate in all kind of hand fracture patterns, even in the most complex of them, which are associated with severe soft-tissue injury. However, an objective evaluation revealed a difference between groups with minor and severe injury. Grip strength and TAM was significantly better in closed fractures in comparison to open fractures. Similarly, extra-articular fractures had superior TAM and grip strength compared to intra-articular fractures, even though this difference did not reach statistical significance. These findings are similar and consistent with the literature, which reveals that the original injury rather than the implant determines the final outcome [5,16-18]. 

Even though the complication rate reached 10% (12 complications in nine of 90 patients), nine of them (skin necrosis and digital nerve trauma) can be attributed to the initial injury and only three of them (3 of 12 complications) are secondary to the use of the implant. Implant removal was required only once because of fracture malunion. Other complications like cold intolerance, re-fracture around the plate, or extensor tendon tethering that have been reported in the literature [15,16], were not observed in this series. Implant removal was not necessary after a long follow-up.

Hand fractures are often fixed with K/Ws, which are considered a minimally invasive and inexpensive procedure but relative unstable, particularly in unstable or complex fracture configuration. The rotational deformity is poorly tolerated in hand fractures Pinning has an advantage in terms of ease of technique and soft tissue sparing, but plating is superior in terms of direct fracture reduction and commencement of early range of motion, without risking the loss of reduction. In metacarpal fractures, the comparison of the two modalities has yielded comparable functional results in the long term, suggesting that an important aspect to consider is the choice of modality according to the skills and preference of the surgeon.

The use of miniature external fixators in a wide range of fracture patterns, especially in open or comminuted ones, is reported to have good to excellent outcomes [7,9]. The disadvantages include difficulty in their use, patient discomfort, increased cost, and the need for taking care of the device in order to avoid pin infection. Their use is limited in the treatment of “the intraarticular and the comminuted fractures where other means of fixation provide a suboptimal result, with usually additional trauma to the soft tissues” [13].

The goal of surgical intervention is an accurate and stable anatomic reduction of the joint and proper restoration of the anatomy of the shaft that enables early mobilization and union of the fracture. The use of plates and screws offers anatomic reduction, fracture compression, increased tensile strength, and preservation of fracture alignment when compared to other available treatments [6,12,18]. This kind of treatment can lead to a reduction of pain and can be combined with early mobilization and enhanced rehabilitation. In addition, the use of plates and screws avoids the danger of pin infection that is anticipated with K/Ws or mini external fixators and makes wound management easier [26]. Although other treatment modalities (such as k-wires or external fixators) of metacarpal and phalangeal fractures were not compared in our study, Başar et al. [21] reached the conclusion that treatment with mini plate plus screws should be avoided in spiral and oblique phalangeal fractures, whereas treatment with mini plate plus screws should be preferred in patients with spiral and oblique metacarpal fractures. 

The earlier plate and screws designs suffered from bulkier constructions and were vulnerable to soft tissue interference. However, the titanium low-profile plates and screws show equivalent strength to 2.7 DCP (dynamic compression plate) plating system [12], have smaller screw sizes suitable for osteosynthesis of small fragments, and do not interfere with soft tissues, enabling improved mobilization. Nonetheless, the low-profile plating system has its own disadvantages. The application of low-profile plates and screws has a steep learning curve and is less forgiving for misplacement. Furthermore, bigger exposure is needed compared to the application of external devices. Additionally, there is a potential risk of fracture beneath the plate secondary to stress shielding and osteopenia, although rare with the new low-profile designs. Finally, the cost of the plates and screws is higher in comparison to K/Ws [18,26].

A newer design with bio-absorbable implants has been utilized recently. Theoretical advantages include increased cost-effectiveness, reduced stress shielding, gradual load transfer, and thus, augmentation of the healing process, no need for implant removal, and no soft tissue interference after their absorption. Nevertheless, inherited disadvantages with their use involve their increased cost, the demanding application with longer learning curves, their reduced strength, and the rapid loss of their original strength that might lead to increased re-fracture rate and the danger of inflammatory reactions [11,14,19,20].

The main disadvantage of the present study is that it does not include a control group with a different fixation technique or with a conservative approach. Other methodological limitations include its retrospective study design and the wide range of fracture types that were studied.

## Conclusions

In conclusion, this study showed that the careful application of titanium, low-profile implants results in satisfactory outcomes even in cases of open or intra-articular fractures and that the original injury defines the long-term result. However, it must be emphasized that the prerequisite for a successful outcome is the meticulous utilization of these technically demanding implants. A variety of fixation methods of hand fractures have been described, the familiarity of the surgeon with each method, and its complications and fracture personality should greatly influence her or his choice.
